# Interprofessional medication assessment among home care patients: any impact on functioning? Results from a randomised controlled trial

**DOI:** 10.1186/s12877-020-01796-1

**Published:** 2020-10-06

**Authors:** K. Auvinen, A. Voutilainen, J. Jyrkkä, E. Lönnroos, P. Mäntyselkä

**Affiliations:** 1 The East Savo Hospital District, BOX 111, FI-57101 Savonlinna, Finland; 2 Institute of Public Health and Clinical Nutrition, Faculty of Health Sciences, University of Eastern Finland, Kuopio, Finland; 3 Assessment of Pharmacotherapies, Finnish Medicines Agency, Kuopio, Finland; 4 Kuopio University Hospital, Primary Heath Care Unit, Kuopio, Finland

**Keywords:** Functioning, Home care, Medication therapy management, Medicines, Older people

## Abstract

**Background:**

Multimorbidity and polypharmacy are related to the use of potentially inappropriate medicines and negative clinical outcomes including drug-related adverse events and functional declines. Home care clients are a vulnerable patient group often exposed to these risks. The aim of this study was to examine whether an interprofessional medication assessment can influence the functioning of home care patients.

**Methods:**

The FIMA study was a randomised controlled intervention study comparing a general practitioner-led interprofessional medication assessment conducted at the baseline of the study with usual care with a six-month follow-up. We used linear mixed models (LMM) with a random subject effect to detect differences between the usual care and intervention groups in the following outcome measures; Katz index of Activities of Daily Living (ADL), Lawton and Brody scale of Instrumental Activities of Daily Living, Timed up and go-test (TUG), Mini-Mental State Examination, Geriatric Depression Scale and the 3-level version of EQ-5D.

**Results:**

Home care patients (*n* = 512) had major disease burdens and functional limitations. Regarding TUG times, the LMM detected a one second improvement in the FIMA group and 2.4 s worsening in the usual care group. However, the result was not statistically significant. The ADL revealed an interaction across time, treatment and sex (*p* = 0.026). The ADL score decreased in both groups; the decline being the steepest among women in the intervention group.

**Conclusions:**

In general, medication assessments may have limited impact on functioning of older people. Nonetheless, the FIMA intervention may prevent worsening of mobility among older home care patients.

**Trial registration:**

The Interprofessional Medication Assessment for Older Patients**,** Clinical Trials.gov. NCT02398812. First registration, 26 March 2015. Retrospectively registered.

## Background

The number of home care patients is increasing rapidly with the ageing of the population. A key requirement for living at home in old age is maintaining physical and psychosocial functioning. Functional declines have been clearly associated with medication-related problems in vulnerable older people [1, 2]. Potentially inappropriate prescribing and drug-related adverse events increase the risks of cognitive impairment, falls and hospital admissions [3, 4].

Several studies have evaluated the impacts of medication assessments in reducing the complexity of medications and inappropriate prescribing [5–7], but less attention has been given to concrete health outcomes such as drug-related adverse events, functioning, general health and quality of life [8, 9]. The scarcity of studies concerning these health outcomes might partly be because it is very challenging to design a practical and feasible medication assessment model for older patients with multimorbidities and being administered polypharmacy.

An interprofessional team method has been suggested as a solution to promote the rational medicine use among older people [10]. The Finnish Interprofessional Medication Assessment (FIMA) is a phyician led, repeatable and pragmatic model for medication optimization of older people [11]. The FIMA model was developed for home care settings. Baseline findings of the FIMA study showed that home care patients had a significant disease burden. The majority of patients (87%) had excessive polypharmacy (≥10 medicines), clinically relevant drug-drug-interactions (74%) and a risk of drug-induced impairment in renal function (85%). Functional limitations including mobility and balance problems were also common in this patient group. In the present study, we examined whether the FIMA intervention exerted any effects on the physical, cognitive, and psychosocial functioning, or the health-related quality of life of home care patients.

## Methods

### Study design and participants

The FIMA study was a randomized, controlled intervention study involving a comparison between a physician led interprofessional medication assessment and usual care in public home care settings. The complete study design of the FIMA study has been published previously [11]. The Research Ethics Committee of Northern Savo Hospital District and Kuopio University Hospital approved the FIMA study protocol on February 3, 2015. The FIMA study was registered with Clinical Trials.gov on March 20, 2015 (identifier: NCT02398812). Reporting follows the CONSORT 2010 statement.

We screened and recruited patients receiving regular home care services in the study areas. The inclusion criteria were age ≥ 65 years and registration to public home care services, and at least one of the following: ≥ 6 medicines in use, dizziness, orthostatic hypotension or a recent fall. We excluded patients whose medication was not managed by the home care and patients undergoing active cancer therapy.

**Fig. 1:**
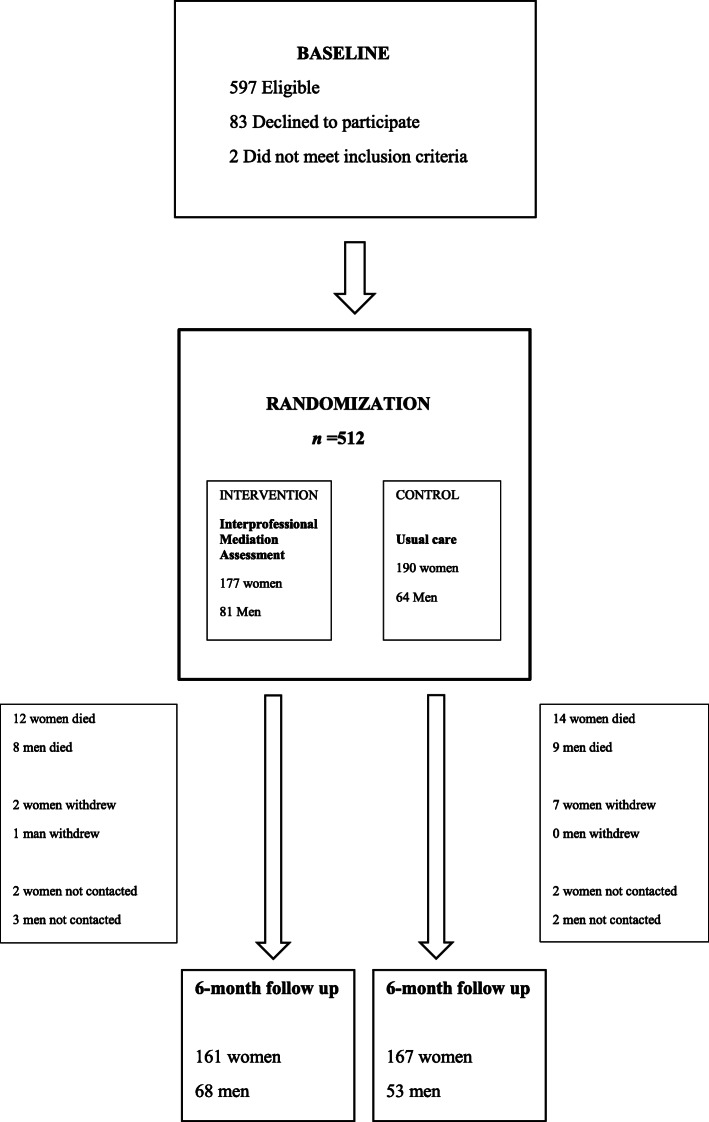
Flow chart of the study

A total of 512 patients were recruited (Fig. 1). The characteristics of the participants have been described previously [11]. Written informed consent was obtained from all individual patients included in the study or their closest proxy if the patient had cognitive impairments. After baseline measurements, patients were randomized to receive the intervention or care-as-usual. Intervention and usual care groups were treated similarly except for the interprofessional medication assessment.

### Data collection

Medication use and patients’ diagnoses were verified according to a structured protocol [11, 12]. Comorbidity was defined according to a modified Charlson Comorbidity Index (CCI) [13]. Performance in daily activities, the patient’s physical and cognitive performance, depressive symptoms and quality of life were assessed. Sociodemographic variables were also collected.

### Intervention

The structured medication assessment included a review of medication, an assessment of the clinical information, and a meeting of an interprofessional team consisting of a pharmacist, physician and registered nurse working regularly in home care; they conducted the medication assessment within two weeks after the baseline measurements. Patients’ updated and verified medication lists, baseline measurements, and electric medical records including their medical histories were available during the assessment.

Before the team meeting, the pharmacist reviewed the patients’ medication lists using four databases: SFINX® (currently INXBASE®) for drug-drug-interactions, PHARAO® (currently RISKBASE®) that complements SFINX® with regard to 11 clinically relevant adverse effects, RENBASE® for renal risks [14] and the Database of Medication for the Elderly (Meds75+) [15]. The physician gathered information from medical records and on the patients’ current clinical status.

The interprofessional team meeting discussed the patient’s current health status and functioning and reviewed the patient’s medications. The physician made clinical decisions and wrote conclusions into the patient’s medical records at the end of the team meeting. The nurse updated the medication lists and informed patient about the changes; if necessary, the patient could participate in the interprofessional team meeting. The average time for the interprofessional team meeting was 20 min, with the structured review done by the pharmacist lasting 27 min.

All pharmacists had a qualification in comprehensive medication review or current continuing professional development in clinical pharmacy. All interprofessional team members undertook a one-day training course or a personal introduction concerning the FIMA protocol.

### Usual care

Patients randomised to usual care did not receive an interprofessional medication assessment. Information on their medication use was collected in a similar manner as in the intervention group but their baseline medication lists were reviewed by a pharmacist only after the six-month measurements had been conducted.

### Outcome measures

Katz index of Activities of Daily Living (ADL) [16] and the Lawton and Brody scale of Instrumental Activities of Daily Living (IADL) scale [17] were used to evaluate each patient’s performance. The maximum score in ADL is six; in IADL it is eight, with lower scores indicating increased requirement for assistance in daily activities. The Timed Up and Go (TUG) test was used to assess mobility, lower extremity strength and balance. The time taken to complete the TUG test correlates with the level of functional mobility [18]. The Mini-Mental State Examination (MMSE) was used for screening cognitive functions; MMSE scores ≤24 indicate impaired cognitive function [19]. The Geriatric Depression Scale (GDS-15) was used for assessing depressive symptoms. Sum scores ≥6 are suggestive of depression [20]. The preference-based, five-dimension instrument provided by EuroQol (EQ-5D-3L)(1) [21] was used for measuring health-related quality of life. These measurements were carried out at baseline and repeated at the six-month follow-up.

### Statistical methods

Data were analysed according to randomisation group irrespective of whether the patients received the intervention as planned (the intention to treat principle). Baseline characteristics of the sample were summarized using proportions, percentages, and means with standard deviation (SD).

We used linear mixed models (LMM) with a random subject effect to detect differences in ADL, IADL, TUG, MMSE, GDS-15, and EQ-5D-3L between the usual care and intervention groups. Treatment (FIMA vs. usual care), time (baseline vs. 6-month follow-up), and sex served as factors, and age and CCI (excluding dementia) at baseline served as covariates. Based on our preliminary analyses (shown as request), the final models also included a treatment-time-sex interaction. IBM® SPSS® Statistics Version 25 served as the statistical platform.

## Results

### Participant characteristics

The mean age of men was 83.1 (SD 6.9) and that of women was 85.2 (SD 6.2) years. Most participants, 64.1% of men and 81.2% of women, were living alone. The mean number of all medicines was 15 in both sexes, ranging from 2 to 36 in women and from 4 to 35 in men. The corresponding numbers for regularly consumed medicines were 9.5 (range: 1–20) in women and 9.6 (3–17) in men. At baseline, there were no statistically significant differences between intervention and usual care groups in either women or men (Table 1).

**Table 1:** Baseline characteristics of the study participants by sex and randomization status (intervention or usual care)

	Women		Men	
	Intervention ***n*** = 177	Usual Care ***n*** = 190	Intervention ***n*** = 81	Usual Care ***n*** = 64
Age (years), *mean (SD)*	85.1 (6.62)	85.1 (5.75)	82.9 (6.59)	83.4 (7.18)
Living alone, *n (%)*	149 (84)	149 (78)	53 (65)	40 (63)
Chronic diseases*, n (%)*				
*Cardiovascular diseases*	164 (93)	174 (91)	72 (89)	60 (92)
*Diseases of musculoskeletal system*	115 (65)	125 (65)	43 (53)	30 (46)
*Diabetes*	63 (36)	66 (35)	28 (35)	26 (40)
*Cerebrovascular diseases*	48 (27)	54 (28)	35 (43)	32 (49)
*Dementia*	62 (35)	62 (33)	23 (28)	14 (22)
*Asthma or chronic obstructive pulmonary disease*	29 (16)	41 (16)	23 (28)	12 (19)
*Psychiatric diseases*	34 (19)	32 (17)	15 (19)	7 (11)
*Cancer*	24 (14)	21 (11)	22 (28)	12 (19)
*Gastrointestinal diseases*	30 (17)	28 (15)	11 (14)	8 (12)
*Neurological diseases*	23 (13)	20 (11)	13 (16)	12 (19)
Charlson Comorbidity Index, *mean (SD)*	3.05 (2.48)	2.65 (2.26)	3.81 (3.09)	3.22 (2.41)
All medicines^a^, *mean (SD)*	15 (5.4)	16 (5.2)	15 (4.8)	15 (4.6)
Regularly taken	9 (3.1)	10 (3.0)	10 (2.9)	10 (3.1)
Taken as needed	3 (2.7)	4 (2.6)	4 (2.9)	3 (2.2)

^a^ Including prescription and over-the-counter medicines

### Impact on functioning and health-related quality of life

**Fig. 2:**
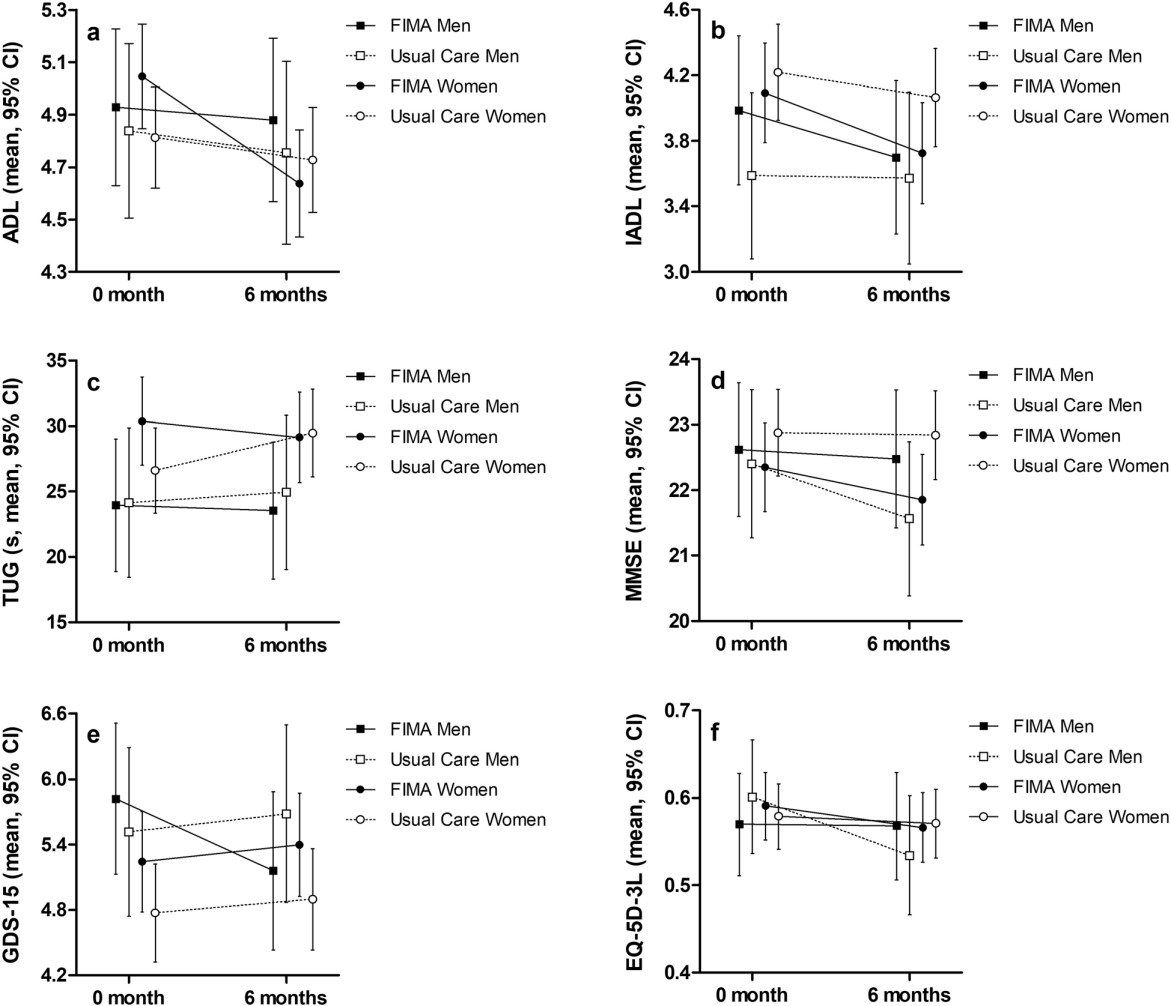
Estimated marginal means of functioning outcomes from Linear Mixed Models. FIMA denotes the intervention treatment. Lowercases refer to functioning measures as follows: **a** Katz index of Activities of Daily Living (ADL), **b** the Lawton and Brody scale of Instrumental Activities of Daily Living (IADL), **c** the Timed Up and Go test (TUG), **d** the Mini-Mental State Examination (MMSE), **e** the Geriatric Depression Scale (GDS-15), and f) the EuroQol health-related quality of life instrument (EQ-5D-3L)

In the usual care group, the TUG time worsened on average by 2.4 s (i.e. the time increased) between baseline and follow-up measurements whereas in the FIMA group, the TUG time improved on average by 1.0 s (Table 2 and Fig. 2). With regard to ADL, the LMM detected a statistically significant time-treatment-sex interaction (Table 2). The ADL score decreased in both groups and both sexes, but the decline was steepest among women belonging to the FIMA group (Fig. 2).

**Table 2:** Functioning at baseline (0 month) and at the six-month follow-up (6 months) together with predicted change in functioning (LMM). In LMM with a random subject effect, treatment (FIMA vs. usual care), time (0 vs. 6 months), and sex served as factors, and age and CCI (excluding dementia) at baseline served as covariates. Values at 0 month and 6 months indicate crude mean ± SD, predicted values indicate mean, 95% CI. *P*-values refer to the treatment-time-sex interaction

	FIMA			Usual Care			P
	0 month	6 months	LMM	0 month	6 months	LMM	
**ADL**	4.98 ± 1.30	4.71 ± 1.49	−0.30, −0.32-(−0.28)	4.82 ± 1.27	4.78 ± 1.37	−0.09, −0.09-(− 0.09)	0.026
**IADL**	4.05 ± 2.01	3.73 ± 2.11	− 0.34, − 0.35-(− 0.34)	4.05 ± 2.06	4.00 ± 2.14	− 0.12, − 0.13-(− 0.11)	0.217
**TUG, s**	28.6 ± 28.2	26.6 ± 22.3	−0.98, −1.03-(− 0.93)	25.5 ± 15.9	26.6 ± 17.7	2.35, 2.24–2.47	0.135
**MMSE**	22.4 ± 4.59	22.2 ± 4.84	−0.39, − 0.41-(− 0.37)	22.7 ± 4.68	22.5 ± 4.64	−0.24, − 0.28-(− 0.20)	0.194
**GDS-15**	5.43 ± 3.20	5.27 ± 3.18	−0.10, − 0.14-(− 0.05)	4.95 ± 3.10	5.00 ± 3.03	0.14, 0.13–0.14	0.121
**EQ-5D-3L**	0.58 ± 0.25	0.57 ± 0.29	−0.017, − 0.019-(− 0.016)	0.59 ± 0.25	0.56 ± 0.27	−0.023, − 0.026-(− 0.020)	0.589

*LMM* Linear Mixed Models, *FIMA* The Finnish Interprofessional Medication Assessment, *ADL* Katz index of Activities of Daily Living, *IADL* Lawton and Brody scale of Instrumental Activities of Daily Living, *TUG* Timed up and go test *MMSE* Mini-Mental State Examination, *GDS-15* Geriatric depression scale, *EQ-5D-3L* Health-related quality of life

## Discussion

We investigated the impact of an interprofessional medication assessment on home care patients’ functioning in a randomised, controlled study design. Based on the mean changes in LMMs, the FIMA intervention appeared to prevent worsening of TUG performance among older home care patients. During the six-month follow-up, functioning as assessed by ADL declined. Instrumental activities, cognitive functioning, mood and quality of life did not exhibit any significant changes during the follow-up. These findings are in line with the results of a systematic review and meta-analysis of randomised, controlled trials involving a medication review as an isolated short-term intervention [22].

In our study, changes in TUG times were different in the intervention and usual care groups favoring the intervention. In general, TUG performance is better among men than women [23] and our results were concordant with this fact. In previous studies, longer TUG times have been associated with lower executive function performance, risk of falls, functional declines and frailty [24–26]. A Canadian study [24] examining older, community-dwelling people, found that the longer it took to complete the TUG test, the greater was the individual’s risk for experiencing a decline in activities of daily living. The three-month risk for a decline in daily functioning rose from 5 to 9-fold when TUG times increased from 20 to 29 s to ≥30 s. In addition, TUG times ≥30 s represented nearly a 4-fold risk for frailty compared with TUG times ≤10 s. Thus, enhancing mobilty and balance perfomance may decrease these risks.

Medication assessments have been examined in several studies in varying settings among older people. A systematic review and meta-analysis of randomised, controlled trials concerning the effectiveness of medication reviews [22] revealed minimal effects on clinical outcomes and no impact on the quality of life. However, the meta-analysis suggested that a medication review may decrease the number of falls, which supports our findings on mobility and balance performance. A Cochrane review concerning pharmacist services for non-hospitalised patients [27] concluded that pharmacist services may slightly improve physical functioning, but physical functioning measured by SPF-36 questionnaire seemed to be enhanced only in diagnosis-specific trials in patients approximately 48–66 years old. For example, two randomised, controlled medication review trials among older patients with multiple conditions found no change in physical functioning [28, 29]. Regarding the present and published findings, it does seem to be difficult to improve physical functioning via an isolated intervention in older people carrying a high disease burden.

The general trend in ADL and IADL functioning is downwards among older people. The clinically relevant change in ADL is considered to be 0.5 or 1.0 point [30] and our intervention could not prevent this decline. ADL scores declined in both groups, and against expectations, even more in the intervention than in the control group. However, the changes in the mean ADL scores were less than 0.5 points.

Our study has several strengths. It was a randomised, controlled study in a real-life context. Furthermore, the study has a practice-based design with a public home care team conducting a pragmatic intervention. In addition, medication assessments are particularly indicated among home care patients. We assume that our findings are generalizable to older home care patients receiving polypharmacy and who are at an increased risk of falls. We used well-known, validated outcome measures to examine different dimensions of functioning. The detection and assessment of medication related risks and interactions were based on four decision support systems that are available and commonly used in the Finnish health care system.

The FIMA procedure has been extensively described and is transferable to different contexts. The procedure has been devised by public health care professionals, which means that information on patient’s clinical conditions is relevantly considered in the medication assessment. Furthermore, the physician can make changes to the patient’s medication at the interprofessional team meeting, when all of his/her information is available. In the FIMA procedure, the home care nurse conducts the verification of the current medication that patient uses at home, which enables medication assessments for large number of patients in routine care. In addition to the records in the patient files, the nurse receives instructions for further follow-up directly at the interprofessional team meeting, which reduces the risks of misinformation and misunderstandings.

This study has some limitations. Due to the high age and multimorbidity, this sample was medically unstable, factors which may diminish the impact of any intervention, especially that of single-domain interventions. We assessed the impact of the medication assessment performed only once, although regular and repeated assessments would be preferable for vulnerable home care patients with changing levels of health and functioning. Nonetheless, it is not recommended to make several changes in older peoples’ medications concurrently. In addition, the same home care teams treated both intervention and usual care patients, which may have caused an observer effect. Although all the interprofessional teams had a one-day training, there might have been some variations in working practices between the teams in the five towns.

## Conclusions

To conclude, it is challenging to improve functioning or prevent functional declines among vulnerable home care patients. Nonetheless, our findings indicate that an interprofessional medication assessment can prevent worsening of mobility of older people receiving home care services.

## Data Availability

The datasets generated and analysed during the current study are not publicly available due national regulations and agreements obtained to perform the study but are available from the corresponding author on reasonable request. The data required to reproduce the present findings cannot be shared at this time as the data also forms part of an ongoing study.

## Abbreviations

FIMA: The Finnish Interprofessional Medication Assessment
ADL: Activities of Daily Living;
IADL: Instrumental Activities of Daily Living
TUG: Timed Up & Go Test
MMSE: Mini-Mental State Examination
GDS-15: Geriatric Depression Scale
CCI: Charlson Comorbidity Index
EQ-5D-3L: EuroQol five-dimension instrument for health-related quality of life
SD: Standard deviation
